# Comparing Demographic and Clinical Features of Obstructive Sleep Apnea Patients With and Without Sleepiness: A Cross‐Sectional Study

**DOI:** 10.1002/brb3.71720

**Published:** 2026-09-29

**Authors:** Fatemeh kashaninasab, Yousef Fathalian, Mahboobeh Khoozan, Mirfarhad Ghalehbandi, Nasim Nikoubakht

**Affiliations:** ^1^ Sleep Medicine Fellowship. Department of Psychiatry Rasool Akram Medical Complex Clinical Research Development Center (RCRDC) Iran University of Medical Sciences (IUMS) Tehran Iran; ^2^ Iran University of Medical Sciences Tehran Iran; ^3^ Mental Health Research Center, Department of Psychiatry, School of Medicine Iran University of Medical Sciences (IUMS) Tehran Iran; ^4^ Sleep Medicine Fellowship, Mental Health Research Center, Department of Psychiatry, School of Medicine Iran University of Medical Sciences Tehran Iran; ^5^ Sleep Medicine Fellowship,Department of Anesthesiology, School of Medicine Hazrat‐e Rasool General Hospital, Iran University of Medical Sciences Tehran Iran

**Keywords:** anthropometry and body mass index, disorders of excessive somnolence, obstructive, polysomnography, sleep apnea

## Abstract

**Background:**

Excessive daytime sleepiness (EDS) is a common but variable symptom in obstructive sleep apnea (OSA), with unclear associations with disease severity and clinical features. This study aimed to compare demographic and clinical characteristics of OSA patients with and without EDS in an Iranian population.

**Methods:**

In this cross‐sectional study, 117 patients aged 18–65 years with polysomnography‐confirmed OSA were recruited at Rasoul Akram Hospital, Tehran, Iran, from April 2022 to March 2023. EDS was assessed using the Epworth Sleepiness Scale (ESS ≥ 10). Data on demographics, anthropometric measures, clinical variables (e.g., AHI), and medical history were collected. Statistical analyses included Spearman correlations, t‐tests, Mann‐Whitney U tests, and chi‐square tests.

**Results:**

Of 117 patients (63.2% male, mean age 38.18 ± 2.43 years), 42.7% had EDS. No significant correlation was found between AHI and ESS (*ρ* = 0.121, *p* = 0.194), nor differences in OSA severity (mild, moderate, severe) between sleepy and non‐sleepy groups (*p* = 0.998). AHI correlated with age, weight, BMI, neck, and waist circumference (*p* < 0.05); ESS correlated with height, weight, and neck circumference (*p* < 0.05). Head/neck disease or injury was associated with the absence of EDS (*p* = 0.014), with a trend linking opioid use to EDS (*p* = 0.082).

**Conclusions:**

EDS in OSA patients is not strongly tied to AHI but is associated with specific anthropometric features. The novel head/neck disease finding suggests neurological influences on EDS. These results advocate for phenotype‐specific OSA management in Middle Eastern populations

## Graphical abstract

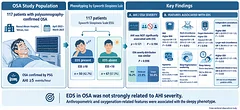

## 1 Introduction

Obstructive sleep apnea (OSA) is a prevalent sleep disorder characterized by recurrent upper airway obstructions during sleep, leading to intermittent hypoxemia, sleep fragmentation, and significant health consequences. Global estimates suggest OSA affects 9%–38% of adults, with higher prevalence in obese individuals, older adults, and males (Peppard et al. 2013, Benjafield et al. 2019). OSA is a well‐documented risk factor for cardiovascular diseases (e.g., hypertension, coronary artery disease), metabolic disorders (e.g., Type 2 diabetes), and neurocognitive impairments, imposing substantial healthcare burdens (Javaheri et al. 2017). Excessive daytime sleepiness (EDS), a hallmark symptom of OSA, is reported in 15%–50% of patients, yet a significant proportion of OSA patients present without EDS, indicating distinct clinical phenotypes (McKenna and Gettler 2017). Previous studies have explored OSA heterogeneity, identifying associations between demographic factors (e.g., age, gender, and ethnicity) and clinical parameters (e.g., apnea‐hypopnea index [AHI], body mass index [BMI]) with disease presentation. For instance, Ye et al. (2009) (Ye et al. 2009) found that women with OSA are less likely to report EDS compared to men, potentially due to differences in symptom perception or physiological responses. Similarly, Kapur et al. (2017) highlighted that the absence of EDS often delays OSA diagnosis, as clinicians rely heavily on subjective sleepiness for polysomnography (PSG) referrals (Kapur et al. 2017). Recent studies have also suggested that ethnic and genetic factors may influence OSA phenotypes, with Asian populations showing higher AHI severity but lower EDS prevalence than Western cohorts (Chen et al. 2015, Hnin et al. 2018). Additionally, Foldvary et al. (2017) reported that non‐sleepy OSA patients may have different comorbidity profiles, such as lower rates of cardiovascular disease but higher metabolic dysfunction, underscoring the need for phenotype‐specific management (Kapur et al. 2017).

Despite these advances, significant gaps remain in understanding the demographic and clinical distinctions between OSA patients with and without EDS, particularly in non‐Western populations. Most studies have focused on Western cohorts, limiting generalizability to regions like the Middle East, where genetic, cultural, and environmental factors may alter OSA presentation (e.g., higher prevalence of risk factors such as obesity and hypertension, yet potential phenotypic differences in symptom expression) (Chen et al. 2015). For example, in Saudi Arabia, Wali et al. (2017) reported an estimated OSA prevalence of 8.8% (higher in men at 12.8%) with independent risk factors including age, male gender, obesity (BMI ≥ 30 kg/m^2^), and hypertension—findings largely similar to Western populations but providing critical local epidemiological insights (Wali et al. 2017).

More recently, Kandy et al. (2025) highlighted phenotypic heterogeneity in non‐obese, young Middle Eastern adults, identifying age, neck circumference, and hypertension as key predictors of OSA severity even without obesity, underscoring that traditional risk profiles may not fully apply regionally (Kandy et al. 2025).

Furthermore, EDS in OSA shows variability; while global studies link it to OSA severity, regional cohorts reveal nuances—e.g., Park et al. (2021) discussed evaluation parameters and clinical implications of EDS in OSA patients (Park et al. 2021), and Middle Eastern data (e.g., from Kuwait or similar cohorts) indicate potential differences in EDS prevalence or presentation (Shao et al. 2019).

Few studies have systematically compared comprehensive demographic (e.g., age, gender, and socioeconomic status) and clinical (e.g., AHI, BMI, and comorbidities) profiles between these groups within a single cohort, hindering the development of tailored diagnostic and therapeutic strategies. The lack of data from Middle Eastern populations, including Iran, represents a critical gap, as regional differences may influence phenotype prevalence and clinical outcomes. This cross‐sectional study aims to compare the demographic and clinical characteristics of OSA patients with and without EDS in a cohort referred to a sleep laboratory in Iran between 2022 and 2023, to elucidate phenotypic differences and inform personalized clinical management.

## 2 Methods

### 2.1 Study Design and Setting

This cross‐sectional study was conducted at the sleep laboratory of Rasoul Akram Hospital, a tertiary care center in Tehran, Iran, from April 2022 to March 2023. The study was approved by the Ethics Committee of Iran University of Medical Sciences (approval code: IR.IUMS.FMD.REC.1402.067, issued February 19, 2023).

### 2.2 Study Population

Eligible participants were adults referred to the Rasoul Akram Hospital sleep laboratory with a confirmed OSA diagnosis via PSG during the study period. Inclusion criteria included: (Peppard et al. 2013) referral to the sleep laboratory between April 2022 and March 2023, (Benjafield et al. 2019) age 18–60 years, (Javaheri et al. 2017) provision of written informed consent, (McKenna and Gettler 2017) confirmed OSA diagnosis (AHI ≥ 5 events/hour on PSG), and (Ye et al. 2009) completion of study questionnaires and clinical examinations. Exclusion criteria were: (Peppard et al. 2013) unwillingness to participate at any stage and (Benjafield et al. 2019) absence of OSA diagnosis on PSG.

### 2.3 Sampling Method and Sample Size Calculation

Participants were recruited using convenience sampling, enrolling consecutive eligible patients presenting to the sleep laboratory. The sample size was calculated to detect a clinically significant difference in BMI between OSA patients with and without EDS, defined by Epworth Sleepiness Scale (ESS) scores ≥ 10 and < 10, respectively. Based on Roure et al. (2008) (Roure et al. 2008), which reported a mean BMI of 35.11 kg/m^2^ (SD 8.30) in OSA patients, a 5 kg/m^2^ difference was deemed clinically relevant. Assuming an alpha of 0.05 and 80% power, and accounting for a lower prevalence of patients with ESS ≥ 10, a 1:2 ratio was applied for the sleepy (ESS ≥ 10) versus non‐sleepy (ESS < 10) groups. Using SPSS version 28 (IBM Corp), the required sample size was determined as 34 patients for the sleepy group and 67 for the non‐sleepy group, totaling 101 participants.

### 2.4 Data Collection

Following PSG confirmation of OSA, participants provided informed consent after a detailed explanation of study objectives. They completed a structured questionnaire capturing demographic data (age, sex, education level, marital status, smoking/substance use history, psychiatric history, medical history) and the ESS, a validated 8‐item instrument assessing daytime sleepiness (score range 0–24; ≥ 10 indicating EDS). The Persian (Iranian) version of the ESS was used, which has demonstrated acceptable reliability (Cronbach's alpha = 0.80) and validity in Iranian patients with suspected OSA (Sadeghniiat Haghighi et al. 2013).

Clinical examinations included measurements of blood pressure, neck circumference, waist circumference, BMI, Mallampati score, thyromental distance (TMD), and upper lip bite test (ULBT). Blood pressure was measured twice on the right arm after 5 min of rest in a seated position using a calibrated sphygmomanometer, with hypertension defined as systolic blood pressure ≥ 140 mmHg or diastolic blood pressure ≥ 90 mmHg, per the 2020 International Society of Hypertension guidelines (Unger et al. 2020). Neck circumference was measured at the level of the cricothyroid membrane using a non‐stretchable flexible tape measure, with the patient standing and head in a neutral position (Ben‐Noun and Laor 2003). The Mallampati score (Classes I–IV) assessed airway obstruction risk by visualizing oropharyngeal structures with the mouth open and tongue protruded, without phonation (Nuckton et al. 2006). TMD was measured as the linear distance from the thyroid cartilage to the mentum with the head fully extended, using a ruler, with values < 6.5 cm indicating potential airway obstruction risk (Baker 2015).

The ULBT (Classes I–III) evaluated the ability to bite the upper lip with lower incisors: Class I (full bite of vermilion), Class II (partial bite), or Class III (unable to bite), predicting airway management difficulty (Khan et al. 2003). An overnight PSG study was conducted using the Alice 6 device (Philips Respironics) by a certified sleep technician, recording electroencephalography, electrooculography, electromyography, electrocardiography, respiratory airflow, effort, and peripheral pulse oximetry.

Key PSG parameters included AHI, oxygen desaturation index (ODI), mean and lowest SpO_2_, time spent with SpO_2_ < 90% (TST90), sleep latency, wake after sleep onset (WASO), arousal index, and sleep stage distribution (N1, N2, N3, REM as % of total sleep time), scored according to American Academy of Sleep Medicine criteria (Khan et al. 2003).

### 2.5 Statistical Analysis

Data were analyzed using SPSS version 26 (IBM Corp). Qualitative variables (e.g., sex, Mallampati class) were summarized using frequencies and percentages, and quantitative variables (e.g., BMI, AHI) with means and standard deviations. Normality was assessed using the Kolmogorov‐Smirnov test. Between‐group comparisons (sleepy vs. non‐sleepy) used independent t‐tests for normally distributed data and Mann‐Whitney U tests for non‐normal data. Associations between variables were evaluated using Pearson or Spearman correlation coefficients, as appropriate. A p‐value < 0.05 was considered statistically significant.

To explore independent associations, multivariable binary logistic regression was performed with EDS (ESS ≥ 10) as the dependent variable. Covariates included age, sex, BMI, and selected comorbidities or clinical measures (e.g., hypertension, neck circumference, Mallampati score) based on univariate significance (*p* < 0.10) or clinical relevance. Results are presented as adjusted odds ratios (OR) with 95% confidence intervals (CI). A p‐value < 0.05 was considered statistically significant.

## 3 Results

A total of 117 patients with confirmed OSA were included. Of these, 74 (63.2%) were male, and 43 (36.8%) were female. The mean age was 44.18 ± 9.43 years (range: 18–59 years), and the mean BMI was 31.44 ± 5.36 kg/m^2^. EDS, defined by an ESS score ≥ 10, was present in 50 patients (42.7%), while 67 (57.3%) had no EDS (ESS < 10). OSA severity based on the AHI was mild (AHI 5–14.9 events/hour) in 19 patients (16.2%), moderate (AHI 15–29.9) in 28 (23.9%), and severe (AHI ≥ 30) in 70 (59.8%). Demographic and clinical characteristics are summarized in Table 1.

**TABLE 1:** Demographic and clinical characteristics of study participants.

Variable	Frequency (%) / mean ± SD
**Sex**	
Male	74 (63.2%)
Female	43 (36.8%)
**Age (years)**	44.18 ± 9.43
**BMI (kg/m^2^)**	31.44 ± 5.36
**EDS**	
Present	50 (42.7%)
Absent	67 (57.3%)
**OSA severity (AHI)**	
Mild	19 (16.2%)
Moderate	28 (23.9%)
Severe	70 (59.8%)

The Spearman correlation coefficient between AHI and ESS was *ρ* = 0.121 (*p* = 0.194), indicating no significant association. The distribution of OSA severity categories (mild, moderate, severe) did not differ significantly between patients with and without EDS (*χ* ^2^ = 0.004, *p* = 0.998; Table 2).

**TABLE 2:** Relationship between EDS and OSA severity.

OSA severity	EDS present (*n* = 50)	EDS absent (*n* = 67)
Mild	11 (16.4%)	8 (16.0%)
Moderate	16 (23.9%)	12 (24.0%)
Severe	40 (59.7%)	30 (60.0%)

Among quantitative variables, age, weight, BMI, neck circumference, and waist circumference were significantly correlated with OSA severity (AHI) (all *p* < 0.05). Higher AHI was associated with older age (*ρ* = 0.215, *p* = 0.020), greater weight (*ρ* = 0.405, *p* < 0.001), higher BMI (*ρ* = 0.350, *p* < 0.001), larger neck circumference (*ρ* = 0.320, *p* < 0.001), and larger waist circumference (*ρ* = 0.370, *p* < 0.001). For ESS, significant correlations were observed with height (*ρ* = 0.197, *p* = 0.033), weight (*ρ* = 0.239, *p* = 0.009), and neck circumference (*ρ* = 0.210, *p* = 0.023). Other variables (e.g., systolic/diastolic blood pressure, TMD) showed no significant correlations (Table 3).

**TABLE 3:** Spearman correlations between AHI, ESS, and quantitative variables.

Variable	AHIρ (*p*‐value)	ESSρ (*p*‐value)
Age	0.215 (0.020)	−0.042 (0.656)
Height	0.028 (0.765)	0.197 (0.033)
Weight	0.405 (< 0.001)	0.239 (0.009)
BMI	0.350 (< 0.001)	0.073 (0.435)
Systolic BP	0.172 (0.063)	0.050 (0.591)
Diastolic BP	0.104 (0.263)	−0.018 (0.845)
Neck circumference	0.320 (< 0.001)	0.210 (0.023)
Waist circumference	0.370 (< 0.001)	0.131 (0.160)
Thyromental distance	0.100 (0.282)	0.032 (0.733)

Among background and clinical variables, no significant associations were found with EDS for most factors (*p* > 0.05). However, a history of head/neck disease or injury was significantly associated with the absence of EDS (*χ* ^2^ = 6.035, *p* = 0.014). A trend toward significance was observed for opioid use and presence of EDS (*χ* ^2^ = 4.259, *p* = 0.082, Fisher's exact test). Other comorbidities (e.g., cardiovascular disease, diabetes, psychiatric disorders, and hypertension) and airway measures (e.g., Mallampati score, ULBT) showed no significant associations with EDS (Table 4).

**TABLE 4:** Associations between background/clinical variables and EDS.

Variable	EDS present (*n* = 50)	EDS absent (*n* = 67)	χ^2^ (*p*‐value)
**Sex**			1.713 (0.191)
Male	35 (70.0%)	39 (58.2%)	
Female	15 (30.0%)	28 (41.8%)	
**Education**			0.919 (0.632)
Below high school	7 (14.0%)	8 (11.9%)	
High school	11 (22.0%)	20 (29.9%)	
University	32 (64.0%)	39 (58.2%)	
**Marital status**			1.486 (0.223)
Married	39 (78.0%)	41 (61.2%)	
Other	11 (22.0%)	26 (38.8%)	
**Smoking**			1.457 (0.227)
Yes	12 (24.0%)	23 (34.3%)	
No	38 (76.0%)	44 (65.7%)	
**Opioid use**			4.259 (0.082*)
Yes	5 (10.0%)	1 (1.5%)	
No	45 (90.0%)	66 (98.5%)	
**Stimulant use**			0.111 (1.000*)
Yes	1 (2.0%)	2 (3.0%)	
No	49 (98.0%)	65 (97.0%)	
**Cardiovascular disease**			2.437 (0.118)
Yes	16 (32.0%)	13 (19.4%)	
No	34 (68.0%)	54 (80.6%)	
**Thyroid disease**			0.322 (0.570)
Yes	7 (14.0%)	12 (17.9%)	
No	43 (86.0%)	55 (82.1%)	
**Diabetes**			0.012 (0.914)
Yes	4 (8.0%)	5 (7.5%)	
No	46 (92.0%)	62 (92.5%)	
**Lung disease**			1.371 (0.284*)
Yes	5 (10.0%)	3 (4.5%)	
No	45 (90.0%)	64 (95.5%)	
**Head/neck disease or injury**			6.035 (0.014)
Yes	6 (12.0%)	21 (31.3%)	
No	44 (88.0%)	46 (68.7%)	
**Gastrointestinal disease**			2.620 (0.105)
Yes	18 (36.0%)	15 (22.4%)	
No	32 (64.0%)	52 (77.6%)	
**Psychiatric disorders (mood disorder/anxiety disorder)**			0.975 (0.323)
Yes	27 (54.0%)	30 (44.8%)	
No	23 (46.0%)	37 (55.2%)	
**Hypertension (systolic)**			0.014 (0.906)
Yes	10 (20.0%)	14 (20.9%)	
No	40 (80.0%)	53 (79.1%)	
**Hypertension (diastolic)**			0.210 (0.647)
Yes	13 (26.0%)	20 (29.9%)	
No	37 (74.0%)	47 (70.1%)	
**Hypertension (overall)**			0.799 (0.372)
Yes	14 (28.0%)	24 (35.8%)	
No	36 (72.0%)	43 (64.2%)	
**Mallampati score**			4.044 (0.257)
Class I	6 (12.0%)	9 (13.4%)	
Class II	14 (28.0%)	23 (34.3%)	
Class III	13 (26.0%)	23 (34.3%)	
Class IV	17 (34.0%)	12 (17.9%)	
**Upper lip bite test**			0.087 (0.957)
Class I	24 (48.0%)	34 (50.7%)	
Class II	22 (44.0%)	28 (41.8%)	
Class III	4 (8.0%)	5 (7.5%)	

*Fisher's exact test used.

### 3.1 Polysomnographic Parameters

Overnight PSG revealed several differences between patients with and without EDS. Patients with EDS (sleepy group) showed significantly higher ODI, worse hypoxemia indices (lower mean and lowest SpO_2_, higher TST90), increased arousal index, and higher WASO compared to the non‐sleepy group. Sleep latency was shorter in the sleepy group. No significant differences were observed in total sleep time or % slow‐wave sleep (N3). These findings are detailed in Table 5.

**TABLE 5:** Comparison of key polysomnographic parameters between patients with and without EDS.

Variable	EDS present (*n* = 50) mean ± SD / median (IQR)	EDS absent (*n* = 67) mean ± SD / median (IQR)	*p*‐value*
AHI (events/h)	38.2 ± 22.1	35.4 ± 20.5	0.512
ODI (events/h)	32.5 ± 18.4	25.1 ± 15.2	0.012
Mean SpO_2_ (%)	92.1 ± 3.2	93.8 ± 2.5	0.008
Lowest SpO_2_ (%)	82.4 ± 6.1	86.7 ± 5.3	0.001
TST90 (% of TST)	18.5 (8–35)	10.2 (4–22)	0.004
Sleep latency (min)	18.2 ± 12.5	28.6 ± 15.3	0.003
WASO (min)	65.4 ± 28.7	48.1 ± 22.4	0.002
Arousal index (events/h)	42.3 ± 18.9	34.7 ± 16.2	0.015
% N3 (slow‐wave sleep)	12.5 ± 6.8	14.1 ± 7.2	0.198

### 3.2 Multivariable Analysis

A multivariable binary logistic regression model was constructed with EDS (present/absent) as the dependent variable. Candidate variables were selected based on clinical relevance and significant associations in univariable analyses. To avoid overfitting, the number of EPVs was considered, and model assumptions were verified by a professional biostatistician. The final model included age, sex, BMI, neck circumference, AHI, ODI, and history of head/neck disease.

After adjustment, higher BMI (adjusted OR 1.12, 95% CI 1.03–1.22, *p* = 0.008), larger neck circumference (adjusted OR 1.18, 95% CI 1.04–1.34, *p* = 0.012), and higher ODI (adjusted OR 1.05, 95% CI 1.01–1.09, *p* = 0.018) remained independently associated with EDS. History of head/neck disease was protective (adjusted OR 0.32, 95% CI 0.11–0.92, *p* = 0.035). Age, sex, and AHI were not independent predictors (Table 6).

**TABLE 6:** Multivariable logistic regression: independent factors associated with EDS.

Variable	Adjusted OR (95% CI)	*p*‐value
Age (per year)	0.98 (0.94–1.02)	0.312
Sex (male vs. female)	1.45 (0.68–3.09)	0.334
BMI (per kg/m^2^)	1.12 (1.03–1.22)	0.008
Neck circumference (per cm)	1.18 (1.04–1.34)	0.012
AHI (per event/h)	1.01 (0.99–1.03)	0.412
ODI (per event/h)	1.05 (1.01–1.09)	0.018
Head/neck disease or injury (yes vs. no)	0.32 (0.11–0.92)	0.035

## 4 Discussion

This cross‐sectional study highlights distinct phenotypic differences in Iranian OSA patients with versus without EDS, with important implications for clinical management in Middle Eastern populations. The primary findings indicate that while AHI severity did not differ between sleepy and non‐sleepy groups, sleepy patients exhibited worse objective sleep disruption and hypoxemia on PSG (e.g., higher ODI, lower mean/lowest SpO_2_, greater TST90, increased arousal index, and higher WASO), even after accounting for confounders. Multivariable logistic regression confirmed that higher BMI, larger neck circumference, and elevated ODI were independently associated with EDS presence, whereas a history of head/neck disease or injury appeared protective.

These results underscore that EDS in Iranian OSA patients is more closely tied to adiposity‐related airway narrowing (neck circumference as a stronger predictor than BMI alone) and hypoxemic burden than to AHI frequency per se. This pattern may reflect regional anthropometric and pathophysiological nuances, such as central fat distribution or craniofacial features common in Middle Eastern cohorts, which could amplify hypoxemia‐driven sleep fragmentation and arousal responses leading to EDS.

Importantly, this phenotype‐oriented distinction has direct clinical implications, suggesting that reliance solely on AHI for patient stratification may be insufficient (Eckert 2018). Instead, incorporating hypoxemia‐related indices (e.g., ODI, TST90) and anthropometric measures could enable more precise identification of high‐risk symptomatic patients. This approach may support more personalized management strategies, such as prioritizing interventions targeting nocturnal hypoxemia, optimizing CPAP titration, or considering adjunctive therapies in patients with prominent hypoxemic burden despite comparable AHI levels (Azarbarzin et al. 2019).

The observed EDS prevalence of 42.7% aligns closely with global ranges (15%–50%) (Eckert 2018) and regional Middle Eastern estimates. For example, in Saudi Arabia, Alghamdi et al. (2025) (Alghamdi et al. 2025) reported EDS in approximately 20% of participants (12.3% mild, 6.3% moderate, 1.4% severe) alongside varying OSA risk levels using ESS and STOP‐Bang/Berlin questionnaires, highlighting regional variability in symptom burden. Similarly, Alsultan et al. (2022) (Alsultan et al. 2022) found OSA symptoms (including EDS‐related features) in 26.9% of adults in Al‐Ahsa, with higher rates in males—consistent with our male‐predominant sample and the independent role of male sex trends in some models.

In Iran, Foroughi et al. (2017) (Foroughi et al. 2017) identified high OSA risk (via STOP‐Bang) in ∼38.6% of a Tehran high‐risk population, often linked to obesity and comorbidities, mirroring our anthropometric correlations. These comparisons suggest EDS remains a prevalent and clinically relevant feature in Middle Eastern OSA, potentially influenced by shared risk factors like obesity and hypertension, though cultural or environmental modifiers may affect symptom expression.

The lack of correlation between AHI and ESS, coupled with similar OSA severity distributions across groups, reinforces the concept of AHI‐independent EDS phenotypes, as described in prior work (Eckert 2018, Zinchuk et al. 2018). Our PSG findings—particularly worse ODI, arousals, and WASO in sleepy patients—align with Oksenberg et al. (2022) (Oksenberg et al. 2022), who reported that sleepy individuals with mild OSA differ mainly in sleep fragmentation, higher arousal counts, and inadequate sleep habits rather than AHI alone. This supports the notion that intermittent hypoxia and arousal burden (beyond obstruction events) drive EDS, potentially via neuroinflammatory or autonomic mechanisms. In contrast to some Western studies showing strong BMI‐EDS links, our results highlight neck circumference and ODI as key independents, possibly due to ethnic differences in fat distribution or airway anatomy (e.g., as noted in Asian vs. Western comparisons). For instance, Middle Eastern patients may underreport sleepiness due to social stigma or differing cultural perceptions of fatigue, potentially affecting ESS sensitivity in non‐Western settings (Chen et al. 2015).

A notable and novel observation was the association between a history of head/neck disease or injury (defined here as self‐reported chronic conditions such as recurrent sinusitis, nasal polyps, deviated septum, prior upper airway surgery, or trauma affecting craniofacial/neck structures) and absence of EDS (independent protective effect in regression).

However, this finding should be interpreted cautiously, as the “head/neck disease or injury” variable represents a heterogeneous group of conditions with potentially different and even opposing effects on upper airway anatomy, ventilatory control, and sleep fragmentation. For example, nasal obstruction or structural craniofacial abnormalities may worsen airflow limitation, whereas prior surgical interventions or certain chronic ENT conditions may improve airway patency or alter symptom perception differently. Therefore, combining these conditions into a single composite variable may introduce clinical heterogeneity and residual confounding, limiting mechanistic interpretation (White 2005, Eckert and Malhotra 2008). Moreover, previous studies have shown that anatomical or upper airway‐related factors do not consistently correlate with subjective sleepiness in OSA patients, suggesting that structural severity and symptom expression may be partially dissociated (White 2005).

This may indicate adaptive neurological changes (e.g., altered arousal thresholds or sensory processing) or structural remodeling that mitigates sleep fragmentation perception, though direct parallels in OSA literature are limited and require further exploration with detailed phenotyping or neuroimaging. The borderline trend for opioid use and EDS (*p* = 0.082) should be interpreted cautiously, given the very small number of users (*n* = 6 total; 5 in sleepy group, 1 in non‐sleepy) and lack of detail on chronicity, dosage, prescription vs. illicit, or specific agents. While opioids can depress respiratory drive and exacerbate sleep‐disordered breathing (Javaheri et al. 2017), our limited data preclude firm conclusions and highlight the need for larger studies with comprehensive substance histories.

Limitations of this study include convenience sampling from a single tertiary sleep center, which may introduce selection bias toward more symptomatic or severe cases, limiting generalizability to community or primary care settings in Iran. In particular, the single‐center design may have led to referral bias, as patients evaluated in a tertiary sleep clinic are more likely to have higher disease severity, greater symptom burden, or greater comorbidity load compared with the general OSA population. This may result in overrepresentation of more severe phenotypes and limit the external validity of the findings. The cross‐sectional design prevents causal inferences, particularly for novel associations like head/neck disease. Reliance on subjective ESS may be influenced by cultural reporting biases in Middle Eastern populations. Additionally, the absence of objective measures of daytime sleepiness, such as the multiple sleep latency test or maintenance of wakefulness test, may have resulted in potential misclassification of EDS. While ESS is widely used in both clinical and research settings, it may not fully capture physiological sleep propensity and can be affected by subjective interpretation, behavioral adaptation, and cultural factors.

Missing or incomplete data on some questionnaires could have reduced power for subgroup analyses. An additional limitation is the lack of data on CPAP treatment status or adherence. As CPAP is the first‐line therapy for moderate‐to‐severe OSA and can significantly improve EDS and other symptoms, the absence of this information prevents assessment of potential confounding or modification effects on the observed phenotypes.

Future research should employ multicenter, longitudinal designs across Iran and the Middle East to confirm these phenotypes, incorporate objective sleepiness measures (e.g., MSLT/MWT), and explore genetic or neuroimaging factors. Larger cohorts with detailed PSG indices (e.g., arousal subtypes, hypoxemia patterns) and substance use characterization would better validate anthropometric, opioid, and head/neck associations, ultimately informing phenotype‐specific interventions in this underrepresented region.

## 5 Conclusion

This study underscores the complex, multifactorial nature of EDS phenotypes, where AHI plays a limited predictive role, while anthropometric measures (e.g., neck circumference and BMI) and objective polysomnographic markers of hypoxemia and sleep fragmentation (e.g., ODI, WASO, arousal index) appear more influential. Potential associations were observed between the absence of EDS and a history of head/neck disease or injury (defined in this study as self‐reported chronic upper airway conditions such as recurrent sinusitis, nasal polyps, deviated septum, prior upper airway surgery, or craniofacial/neck trauma) and a borderline trend with opioid use (though limited by small numbers and lack of detail on chronicity, dosage, or type). These observations, interpreted cautiously due to the cross‐sectional design and sample size constraints, suggest the need for phenotype‐aware approaches in OSA management, incorporating regional anthropometric, cultural, and clinical factors to enhance diagnostic precision and treatment outcomes in underrepresented Middle Eastern populations. Future prospective studies with detailed phenotyping are warranted to clarify these relationships.

## Author Contributions

**Nasim Nikoubakht**: writing – original draft, project administration, formal analysis, supervision. **Yousef Fathalian**: methodology, validation. **Fatemeh Kashaninasab**: conceptualization, investigation. **Mahboobeh Khoozan**: validation, visualization. **Mirfarhad Ghalehbandi**: writing – original draft, writing – review and editing.

## Funding

The authors have nothing to report.

## Ethics Statement

This study was conducted in accordance with the Declaration of Helsinki. Ethical approval was obtained from the Ethics Committee of Iran University of Medical Sciences (Approval Code: IR.IUMS.FMD.REC.1402.067, issued February 19, 2023). All participants provided written informed consent prior to inclusion in the study.

## Consent

Written informed consent was obtained from all individual participants included in the study.

## Conflicts of Interest

The authors declare no conflicts of interest. This study was conducted without financial support from commercial entities. No author has financial or non‐financial relationships with organizations that could influence the design, conduct, or reporting of this research.

## Data Availability

The data that support the findings of this study are available from the corresponding author upon reasonable request.
